# Fitness to drive in seizure and epilepsy: A protocol for Iranian clinicians

**Published:** 2019-10-07

**Authors:** Nasim Tabrizi

**Affiliations:** Department of Neurology, School of Medicine, Mazandaran University of Medical Sciences, Sari, Iran

**Keywords:** Seizures, Epilepsy, Drive, Clinical Protocol, Iran

## Abstract

Driving restriction is a well-known undesirable consequence of epilepsy and causes significant problems regarding independence and employment for epileptic patients. Many countries all over the world have provided comprehensive protocols in this regard with the aim of providing the possibility of less restricted, but safe driving for epileptic patients and also providing the opportunity for uniform decision-making for clinicians. However, the available fitness to drive protocol in Iran still lacks sufficient details and clinicians might encounter serious problems in terms of the driving issue in epileptic patients. In order to provide a uniform protocol containing adequate practical data, a systematic review of literature addressing guidelines about driving and epilepsy and driving laws of different countries for epileptic patients was performed and, after consideration of cultural issues, a practical protocol for Iranian neurologists was suggested.

## Introduction

The negative impact of seizures on the quality of life (QOL) of epileptic patients has been acknowledged. Seizures particularly influence the social aspects of life including work, driving, interpersonal relations, and education.^1^^-^^10^ One of the most restricting factors of epilepsy is prohibition of driving that might have adverse effects on QOL, independence, and working capabilities.^11^^-^^15^ The main rationale behind limited rules for driving in epileptic patients is obviously the risk of occurrence of seizure during driving that might lead to significant injuries in patients and others. The first traffic accident due to a seizure has been reported in 1906,^16^ and for a few decades after that, a permanent driving ban law was enforced for all epileptic patients.^17^ Gradually, with the initiation of diagnostic advances and more common use of first antiepileptic drugs (AEDs), the conditional driving license in certain circumstances was allowed and some countries started to provide a comprehensive protocol to cover different types of seizures with the aim of providing the possibility of less restricted, but safe driving for epileptic patients and similar decision-making about driving issues.^18^^-^^21^ However, some other countries still have restricted rules that prevent epileptic patients from driving.^22^

The current evidence shows that the traffic accident rate among epileptic drivers is not as high as what was previously expected^23^^-^^25^ and it might cause a lower rate of morbidity and mortality in comparison to traffic accidents that occur due to heart attack or drinking.^18^ However, there are reports of higher incidence of car accidents^26^^-^^31^ and fatal crashes^32^ in epileptic patients in other studies and there is too much variation in the current evidence to make a certain conclusion.^33^ Many factors influence driving competency in epileptic patients^34^ and due to the variety in types of seizures and epilepsy, patients' risk factors and clinical condition, treatment approaches, compliance to medications, cultural and safety issues, and many other intervening factors, no universal legislation can be prepared yet and each country adheres to its own rules. However, in Iran, no comprehensive protocol exists regarding driving in epileptic patients. This deficiency prevents similar decision-making for clinicians and sometimes might cause undesirable deprivation of social rights for patients. Referring to article 23 of the driving law in Iran, if a person definitely has epilepsy or madness, he/she cannot receive a driving license.^35^ This law is re-emphasized in clause 18, appendix 2 of the latest Iranian law on driving.^36^ The only exceptions are epileptic patients whose seizures are under control through the use of AEDs. For these patients, a 1-year conditional driving license might be issued 3 years after military exemption.^35^ Due to lack of adequate practical data and absence of a uniform protocol, a systematic review of literature addressing guidelines about driving and epilepsy and also driving laws of different countries for epileptic patients was performed, and after consideration of cultural issues, a practical protocol was suggested for Iranian neurologists. 

**Data sources and search strategy**

A comprehensive search of published literature was done in 2000-2017 using PubMed, Embase, and Cochrane databases. The keywords included MeSH and free terms, seizure, epilepsy, driving, guideline, protocol, and Iranian. Fitness to drive protocols were sought separately from driver licensing authorities and epilepsy societies all over the world. In order to find information about the current status of and laws for epileptic drivers in Iran we also reviewed Iranian databases including SID, Magiran, Irandoc, and Civilica with the Persian equivalents for the words Iranian, driving law, seizure, and epilepsy. The focus group was constructed as a mini-group including 3 expert neurologists with long term experience on epilepsy and an epileptologist as moderator. Recommendations were reached by consensus of all members on available evidence in literature and were modified based on our clinical experience and cultural considerations. The final draft of the manuscript was approved by all members. 

**Study selection**

The abstracts were evaluated and relevant information was determined. The reference lists of relevant papers were hand-searched for further relevant literature. The selected abstracts underwent full-text review. The English studies that provided useful data for decision-making about driving in patients with seizure or epilepsy were included. Available consensus guidelines were also considered. Publications in other languages were only contemplated if they had an English abstract with adequate information. In order to provide a comprehensive national protocol, we also considered the Persian literature to find out if there is any previous protocol or helpful data about driving in Iranian epileptic patients.

**Figure 1:**
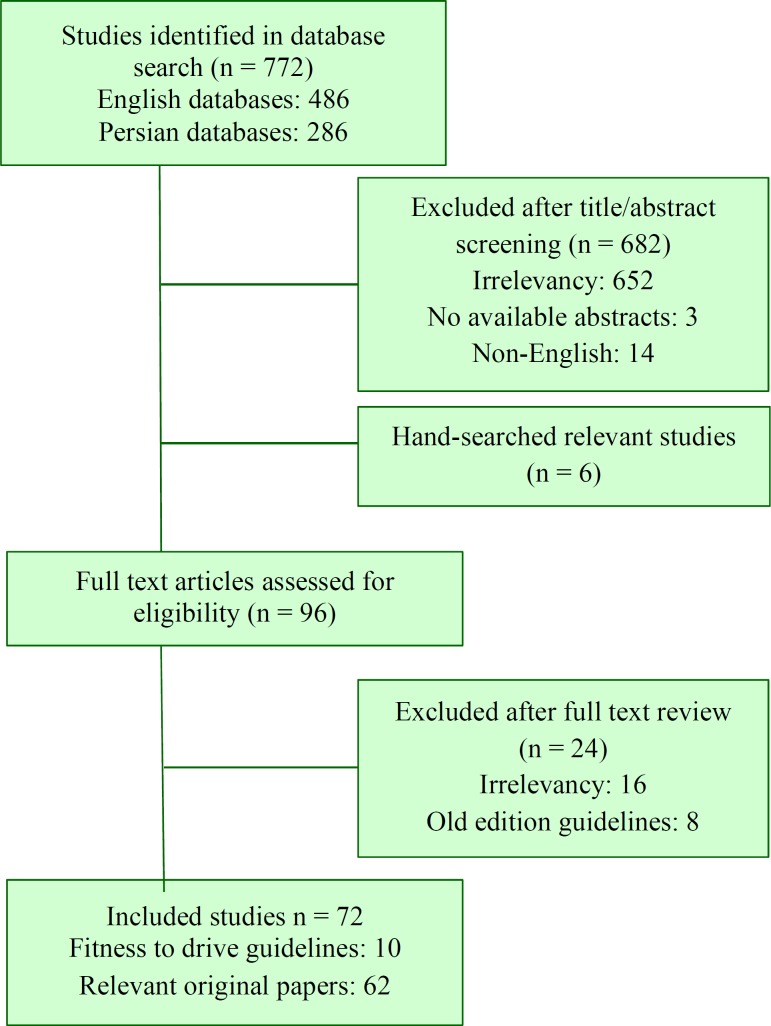
Data gathering process

The data gathering process is provided in figure 1.

**Definitions**

Private drivers were defined as drivers holding a license for a car or motorcycle and not authorized to use the vehicle for carrying public passengers, carrying dangerous goods, or as a driving instructor.^37^

Based on the Iranian road transport authority (IRTA) guideline,^38^ the term commercial driver refers to:

1. Drivers of passenger vehicles including bus, midibus (maximum 34 passengers), minibus (maximum 21 passengers), van (maximum 15 passengers), and car (maximum 4 passengers)
2. Truck drivers including pickup, light truck, and truck.

In this paper, I addressed commercial drivers as drivers carrying public passengers (bus and taxi drivers, chauffeurs, drivers of hire cars, and others as mentioned in the first group of the IRTA guideline), drivers of heavy vehicles (including those mentioned in the second group of the IRTA guideline), and drivers carrying dangerous goods.^37^

The expert opinion and guidelines regarding fitness to drive are different between countries and even states.^39^^-^^45^ However, despite several factors intervening in legislation on driving of epileptic patients, an annual seizure risk of 20% including 50% for private drivers and 1%-2% for commercial drivers are generally acceptable to allow driving.^37^^,^^46^^,^^47^ Thus, regarding safety issues, more strict rules have been approved for commercial drivers.^37^

There is also a general agreement that once a seizure occurs, the driving license is suspended and a defined seizure free period (SFP) should elapse before issuing a new license. The logic behind this agreement is that, the cumulative probability of remaining seizure-free significantly decreases as more time passes from the last seizure.^48^^-^^50^

In the following sections, different situations that clinicians might encounter and different existing protocols about them have been discussed, and recommendations have been provided for Iranian epileptic drivers.

**Drivers with a history of febrile seizures or benign childhood epilepsy syndromes**

History of febrile convulsion or benign childhood epilepsy syndromes have been less considered in fitness to drive guidelines. The main reason for this is that the majority of these diseases are self-limiting and often cease before the usual age of license application. The New Zealand Transport Agency^51^ suggested that childhood febrile convulsions, which stopped before the age of 5 years, is not considered as a history of epilepsy and cause no limitation for license holding. The Australian fitness to drive guideline (Austroads)^37^ recommended that a history of a benign seizure or epilepsy syndrome limited to childhood not be an inhibiting factor for holding an unconditional license for private or commercial drivers, if no seizures have occurred after the age of 11. However, if the patient has experienced a seizure after 11 years of age, the approach is different based on the group the criteria of which the patient meets. Moreover, it has been postulated that if the last seizure has occurred more than 5 years ago, a new seizure could be approached as a first unprovoked seizure.^52^^,^^53^

***Recommendation:*** Patients with a history of febrile convulsion before 5 years of age or benign childhood epilepsy syndromes, which are self-limiting or have been cured at least 5 years before applying for a driving license, may hold an unconditional license.

**First seizure**

Since the recurrence risk following the first seizure is about 40%-50%, driving restriction for a variable period thereafter is commonly recommended by several authorities.^37^^,^^51^^,^^54^^,^^55^ The recurrence risk in the first year decreases significantly with increasing seizure-free interval. Bonnett et al. have reported that while a patient is on AED, after a seizure-free period of 6 months, the overall recurrence risk in the next year will be reduced to less than 20% (14% after 6 months and 7% after 1 year).^56^ Brown et al. found that non-driving periods of 8 months for the first unprovoked seizure and 5 months for the first provoked seizure are needed to achieve a risk of seizure recurrence of 2.5% per month and a monthly risk of a seizure while driving of 1.04 per thousand along with an accident risk ratio of 2.6 (driving time of less than 1 hour per day).^55^ However, considering safety issues, variable periods of driving prohibition have been specified by different jurisdictions.

**First symptomatic seizure**

Acute symptomatic seizure (ASS) refers to a seizure that occurs concurrent with a systemic disorder or in close association with a certain brain injury.^57^ The following classification is practical in defining ASSs of different etiology. The diagnosis of ASS should be considered if seizure occurs within 1 week of traumatic brain injury, stroke, hypoxic encephalopathy, or intracranial surgery, during the first week or in the active phase of a central nervous system (CNS) infection, as presenting symptom or during the first week of relapse in multiple sclerosis, during activation of an autoimmune disease, at first presentation of subdural hematoma, within 24 hours of a severe metabolic derangement, 7-48 hours after last alcohol drink, and a variable time after benzodiazepine and barbiturate withdrawal and certain drug abuses.^57^

The risk of recurrence following ASSs is different from unprovoked seizures, and is estimated to be within the range of 3%-10%.^47^ Thus, ASSs often require long-term treatment and their effect on driving capabilities is generally considered separately.

***Private drivers:*** The Canadian guideline^58^ has classified ASSs into 3 groups. The first group includes provoked seizures caused by a structural brain disease such as brain tumor, aneurysm, stroke, or subdural hematoma. If these patients have a 6-month SFP and the provoking factor is stabilized, resolved, or corrected, with or without treatment, making further seizures unlikely, re-licensing should be considered. The second group contains those who experienced ASS in the setting of a toxic illness, adverse drug effect, trauma, or other etiologies that are not caused by a structural brain disease. These drivers are eligible for a license without delay for a SFP if they meet the criteria mentioned for the first group. The last group includes those with alcohol-related provoked seizures. This group could be eligible for a conditional license after a 6-month SFP if the etiology of the seizure was certainly alcohol use and they undergo addiction treatment and receive a certification of no alcohol abuse or dependency. The Austroads^37^ suggested that for private drivers without a previous history of seizures and in whom seizures occurred only during a temporary brain disorder or metabolic condition (including head injuries and drugs or alcohol withdrawal), the best approach is to issue a conditional license after a 6-month SFP with at least annual recheck. The American Association of Motor Vehicle Administrators (AAMVA) and Federal Motor Carrier Safety Administration (FMCSA)^49^ and the Driver and Vehicle Licensing Agency (DVLA) of the United Kingdom ^59^ recommended the same SFP in alcohol or drug induced seizures unless the patient meets the criteria for substance abuse or dependence in which case they must cease driving until in prolonged remission. Some experts believe that if seizures are certainly a result of provocative conditions, such as sleep deficiency, alcohol use, acute disease, fever, intoxication or metabolic disorders that have been resolved, the SFP before re-licensing could be shortened to 3-6 months.^60^

***Recommendation: ***All patients should undergo full neurological evaluation and if the diagnosis of ASS is definite, patients whose seizures are provoked by temporary brain or metabolic disturbances must refrain from driving for at least 6 months and if they have no additional seizures, conditional driving license with at least an annual review could be issued. If the provoking factor is drug or alcohol, they should desist from driving for at least 6 months, and if they meet the criteria for substance abuse or dependence, they should complete an acceptable rehabilitation program. If substance-screening tests are negative and seizures do not recur, a yearly conditional license may be considered and repeated. 

***Commercial drivers:*** According to the FMCSA guideline,^47^ when ASSs are the result of provoking factors with a low risk of recurrence (including lidocaine-induced seizure, mild concussive seizure, seizure due to syncope, acute metabolic derangement, and drug withdrawal) that did not cause a potentially epileptogenic lesion, driving restriction is not reasonable. However, in conditions with moderate to high risk of additional seizures, such as severe head trauma, intracranial hemorrhage of any cause, brain infection, stroke, and brain tumor, greater care should be taken to certify the ability of the patient to drive. 

The recommended SFP by Austroads^37^ is 1 year with at least 1 electroencephalogram (EEG) without epileptiform activity in the last 6 months and no other epileptiform finding in any other EEGs performed in the last year. The FMCSA guideline^47^ suggested a similar approach to private drivers if seizures are induced by alcohol or drugs. However, the DVLA^58^ presented more severe limitations and stated that following a solitary alcohol-related seizure, the license must be revoked for a minimum of 5 years and license restoration after that requires normal cerebral imaging, no AED consumption for at least 5 years, and maintained abstinence from alcohol if previously dependent and confirmation by an addiction specialist and neurologist.

***Recommendation: ***All patients should undergo a complete neurological evaluation, and if the diagnosis of ASS is definite, patients whose seizures are provoked by temporary brain or metabolic disturbances must refrain from driving for at least 1 year and if they have no additional seizures, cerebral structural disease, epileptiform finding in EEG, and the second seizure is unlikely, conditional driving license with at least an annual recheck could be issued. If the provoking factor is drug or alcohol, they should desist from driving for at least 1 year, and if they meet the criteria for substance abuse or dependence, they should complete an acceptable rehabilitation program. If substance-screening tests are negative and seizures do not recur, a yearly conditional license may be considered.

**First unprovoked seizure**

Unprovoked seizures are defined as seizures occurring without correlation to a potentially responsible clinical problem or after the expected interval for occurrence of ASS.^57^ If more than 1 seizure occurs within 24 hours, they are treated as a single event. Some authorities believe that for private drivers if the interval between the last seizure and penultimate seizure is more than 5 years, the last seizure could be approached as a first unprovoked seizure.^52^^,^^53^ It has been postulated that if patients have been seizure free for at least 4 years following a single unprovoked seizure, the annual recurrence risk might be ≤ 2% thereafter.^47^

***Private drivers: ***The Canadian guideline^58^ recommended that complete neurological assessment be performed to determine the etiology of the seizure, and if epilepsy is not the diagnosis and there is no relevant abnormal findings in CNS imaging and EEG results, the driving license could be renewed after 12 months while reassessment is recommended 1 and 5 years later if no new seizures occur. The Korean guideline also considered 12 months of lack of driving.^61^ However, the consensus for SFP in the other 4 legislations is 6 months.^37^^,^^52^^,^^53^^,^^59^ Most US states required a SFP of 3, 6, or 12 months, whilst others employed case-by-case decisions.^62^ Other jurisdictions have cautiously considered a 5-year SFP before a new license issue.^49^^,^^51^

***Recommendation: ***If complete neurological assessment is suggestive of first unprovoked seizure (non-compatible with diagnosis of epilepsy), reassessment for a new license can be performed 6 months later.

***Commercial drivers:*** The general agreement for this group of patients is a 5-year SFP without AED use^52^^,^^53^^,^^59^, or despite it^37^ if they have undergone complete neurological assessment and there are no clinical findings or paraclinical results such as EEG or brain imaging which indicate an annual recurrence risk of more than 2%. The medical expert panel of the FMCSA^47^ recommended a SFP of a minimum of 4 years before license renewal, and the utilization of a stable medication regimen for the last 2 years if the patient is on AEDs. Contrary to the stricter guideline by other countries, Canada suggested that a 12-month SFP accompanying normal EEG and imaging and yearly reassessment would be satisfactory.^58^

***Recommendation:*** If complete neurological assessment is suggestive of first unprovoked seizure (non-compatible with diagnosis of epilepsy) and EEG and imaging have shown no risk factor for recurrence, reassessment for a new license can be performed 5 years later and annually thereafter.

**Diagnosis of epilepsy**

Regarding the last definition of epilepsy,^63^ if the risk of the second seizure in the next 10 years is ≥ 60%, the diagnosis of epilepsy should be considered. Different types of epilepsy might cause variable kinds of deficits in skills necessary for driving. As indicated in previous studies, seizures that lead to decreased level or content of consciousness during ictal or postictal phases increase the risk of accidents. Thus, primary and secondary generalized seizures and focal seizures with impaired awareness are the types with more significant risks.^64^ There are some other types of seizures with less, but still considerable risk of accidents. Focal seizures with retained awareness might cause involuntary tonic contractions or clonic movements and myoclonic jerks that could lead to unwanted motions. Previous studies have reported that the presence of aura before loss of consciousness could act as a warning sign for the driver and lower the risk of possible accidents.^64^^,^^65^ However, more recent studies have shown less promising results for the protective role of auras in crashes.^66^^,^^67^

The common point between different legislations is that once the diagnosis of epilepsy is established, the permission to drive depends strongly on the patient’s cooperation including complete drug adherence, reporting of new seizures, and regular neurological assessments. In the following sections, the suggested recommendations in different types of epilepsy and their treatment are investigated.

***Private drivers:*** The Austroads^37^ indicated that in patients who had begun treatment with AED for the first time within the past 18 months (new cases of epilepsy), a conditional license and annual reassessment may be considered if the patient has been treated for at least 6 months without any seizures, or if any seizures did occur after treatment, they occurred only in the first 6 months. Other jurisdictions have defined a wide range of SFP including 6 months,^58^ 6-12 months,^51^ 1 year,^52^^,^^53^^,^^61^ and 2 years.^49^ The DVLA^59^ recommended that, if the patient satisfies the regulation, a 3-year license be issued and, if no seizures or disqualifying conditions occurred for 5 years (with medication if necessary), the ’til 70 years license be restored. 

***Recommendation:*** In the case of complete neurologic assessment, good adherence to AEDs, and regular visit to a neurologist, a 6-month SFP is suggested before issuing a new license.

***Commercial drivers:*** Considering patient’s cooperation and satisfying findings on EEG, the recommended time interval before license renewal is generally 5^49^^,^^58^ and 10 years (without use of AED^52^^,^^53^^,^^59^ or despite it^37^). The FMCSA guideline^47^ suggested a SFP of at least 8 years considering that a stable medication regimen for at least 2 years is required if the patient is on AED. However, in New Zealand, such patients are usually regarded as permanently unfit to drive unless they have a 5-year SFP without medication and a neurologist-supported claim.^51^

***Recommendation:*** In the case of complete neurologic investigation, good adherence to AEDs, and regular visit to a neurologist, a 10-year SFP along with an annual assessment is suggested before considering the issuing of a new license.

**Seizures in previously well-controlled epilepsy**

A breakthrough seizure is defined as the first seizure after at least a 1-year SFP in epileptic patients on AED. Based on the SANAD study, the overall recurrence risk of seizure 12 months after a breakthrough seizure is less than 20% if no seizure occurs in this period. However, in patients requiring polytherapy to achieve the initial 12-month remission or those who take at least 2 years to achieve the initial 12-month control of seizures, more than 12 months of SFP is needed for their risk of recurrence to be less than 20%.^46^ Uncontrolled epilepsies are obviously restricted from licensure.^68^

***Private drivers:*** The Austroads recommended the consideration of a conditional license after 4 weeks in the case of a breakthrough seizure as a result of a known provoking factor that could certainly have been avoided and has not caused any seizures before. Nevertheless, if no cause was identified for the seizure, a SFP of at least 3 months in a cooperative patient is required.^37^ If 1 or more seizures have occurred during the last 12 months leading up to the last seizure, the least recommended SFP should be 12 months.^37^^,^^58^ The guideline provided by the New Zealand Transport Agency however addressed the previously defined breakthrough seizure as uncontrolled epilepsy and has postponed the decision-making to when a new treatment approach is used and its efficacy and the patient's adherence is certainly confirmed.^51^ Other guidelines have considered the previously mentioned SFP for patients upon epilepsy diagnosis.

***Recommendation:*** In a patient with previously well-controlled seizure, occurrence of an unprovoked seizure might lead to a 6-month abstinence from driving. If more than 2 seizures occur in a 12-month period, a complete approach consisting of treatment modification, investigation of the patient's compliance, and reassessment for driving fitness after 12 months should be considered.

***Commercial drivers:*** For commercial drivers, Austroads has presented an approach similar to that of patients with newly diagnosed epilepsy (10-year SFP plus fulfilling EEG criteria)^37^ and although not directly indicated, it seems that other guidelines also consider the SFP of patients with a diagnosis of epilepsy in these circumstances.

***Recommendation: ***In this group of drivers, if any unprovoked seizure occurs, complete neurologic reassessment including a possible new treatment approach and investigation of the patient's adherence to AEDs should be considered and at least a 10-year SFP with annual assessment after that seems reasonable.

**Safe seizures**

***Private drivers: ***Despite the controversial essence of the term "safe seizures", Austroads has used it for seizures that do not result in impaired consciousness or the complete ability of the driver to control the vehicle.^37^ According to the guideline, a seizure is considered safe if normal responsiveness has been checked by a reliable witness or video-EEG monitoring. These patients are eligible to drive if the only type of seizure they have experienced during the previous 2 years has been safe seizures.^37^^,^^61^ Two other guidelines directly addressed simple partial seizures that do not impair driving safety (no impairment of consciousness and head and eye deviation^58^) and considered license renewal if the seizure pattern has been consistent for at least 1 year.^58^^,^^69^ Other guidelines have not clearly addressed these kinds of seizures^47^^,^^49^ or have treated them like a diagnosis of epilepsy.^51^^,^^59^

***Recommendation:*** If habitual seizures can be reviewed using video-EEG monitoring which convinces the neurologist that they might not cause any problems in the abilities needed for driving and if they are the only type of seizures experienced during the last 2 years, a new license with annual reassessment can be issued. If the patient does not meet each of the above conditions, a SFP of at least 6 months is recommended before license renewal.

***Commercial drivers:*** The Canadian guideline indicates that patients with simple partial seizures that do not impair driving safety are eligible for driving if it has been 5 years since their last seizure or the seizures are uncontrolled, but their pattern has been consistent for 3 years.^58^ Austroads however followed the standard approach of 10-year SFP with the previously mentioned EEG criteria^37^ and the European guideline has banned driving for these patients.^53^

***Recommendation:*** Due to the importance of safe driving by commercial drivers, a 10-year SFP is suggested before considering a new license issue.

**Sleep-related epilepsy**

Epileptic seizures frequently manifest during the sleep state, but some certain types of them have a specific relation to sleep. These are often classified as pure sleep epilepsies (benign childhood epilepsy with centrotemporal spikes, and sleep-related hypermotor epilepsy), sleep-accentuated epilepsies (Lennox-Gastaut syndrome, syndromes with electrical status epilepticus in sleep), and arousal epilepsies (juvenile myoclonic epilepsy, generalized tonic-clonic seizures upon awakening).^70^ According to the definition provided by the International League Against Epilepsy (ILAE), sleep seizures are seizures that occur exclusively or predominantly (more than 90%) during sleep.^71^ D’Alessandro et al. have reported an estimated risk of 13% for awake seizure in patients with sleep-related epilepsy (SRE) during a 6-year follow-up period.^72^ However, the risk is dramatically reduced to 6.5% in patients who do not have episodes of sudden treatment withdrawal and a high frequency of seizures at inclusion.^72^

In this review, SRE has been addressed as seizures occurring exclusively in sleep (pure SRE). In patients with pure SRE or arousal epilepsies, the risk of awake seizures is low enough to not be considered a hazard for driving.^37^ Therefore, in some guidelines, the option to provide more freedom for patients with uncontrolled pure SRE have been considered. 

***Private drivers:*** Austroads recommended that a new conditional license be considered if none of the previous seizures occurred in a conscious state and the first sleep-only seizure was at least 1 year ago. However, if there have been previous awake seizures, but not in the previous 2 years, the consistent pattern of sleep-only seizures should have existed for at least 2 years.^37^ DVLA and New Zealand Transport Agency guidelines suggested the same approach for patients with the first sleep-only seizure and diagnosis of epilepsy and added that patients may be licensed if attacks have occurred in sleep without any seizure during wakefulness for 3 years.^51^^,^^59^ Nevertheless, if any attack has occurred during wakefulness, a 3-year license issue is dependent on treatment compliance and the certainty that driving is unlikely to impair public safety.^59^ Other guidelines have recommended license renewal if a consistent pattern of sleep-only seizures has existed for at least 1^58^ or 2 years.^61^^,^^69^

***Recommendation:*** If the patient has a consistent seizure pattern of pure sleep or upon awakening types for at least 3 years and the seizures cause no prolonged postictal impairment in wakefulness, reassessment for a license should be considered. 

***Commercial drivers:*** In contrast to private drivers, most guidelines treat commercial drivers as patients with a diagnosis of epilepsy and do not accept any privileges for them.^37^^,^^49^^,^^52^^,^^53^ The only exception is the Canadian guideline which indicated that drivers who have uncontrolled seizures, but their seizure pattern has been consistent in the past 5 years and does not cause prolonged postictal impairment in wakefulness, are eligible for license issue.^58^

Recommendation: Due to the importance of safe driving by commercial drivers, a 10-year SFP and annual assessment thereafter is suggested before considering a new license issue.

**Planned tapering or withdrawal of one or more** **‎** **AEDs** **‎**

Tapering and withdrawal of AEDs might be considered under several conditions such as prolonged seizure control, modification of treatment regime, and drug side effects.^73^^,^^74^

Bonnett et al. have reported that the risk of seizure recurrence in the next 12 months immediately following AED withdrawal and 3 months after that is 30% and 15%, respectively. If seizures recurred and AEDs were reinstated, the annual risk of recurrence 3 and 6 months after resumption of previous AEDs is 26% and 18%, respectively.^75^ The risk of recurrence after AED withdrawal is higher in the first 6 months and, if no seizure occurs in this period, the risk after 1 year is almost the same as that in individuals who continue treatment.^76^

***Private drivers: ***The Austroads^37^ recommended that patients refrain from driving during the tapering period and 3 months after withdrawal. The only exception that needs no driving restriction is when the dose reduction is due to the occurrence of dose-related adverse events and is unlikely to affect seizure control. However, if seizures recur, the last effective medication regime should be resumed and, if no seizures occur during the following 4 weeks, a conditional license can be issued. If seizure recurrence is not observed, the person may become eligible for an unconditional license. The European guideline suggested a driving restriction during the withdrawal period and 6 months thereafter with recommendation of a 3-month SFP if seizures recurred and previously effective treatment is reinstated.^53^ Other guidelines indicated a similar approach with different SFPs of 3,^52^^,^^58^ 6,^59^ 12,^51^^,^^61^ and 24^49^ months if seizures recurred. DVLA also stated that when a change from one medication to another is intended, the efficacy of AEDs should be considered and if the new medication is less efficacious than the previous one, 6 months off driving starting from the end of the changeover period is reasonable.^59^

***Recommendation:*** The patients should not drive during the tapering period and 6 months after withdrawal. If seizures recur, a 3-month SFP after resuming the previous therapeutic regime should be considered. Patients who experience side effects from AEDs should refrain from driving during dose reduction of the related AED, until the side effects improve. When changing from a medication to another, a 6-month SFP after withdrawal from the first drug is recommended.

***Commercial drivers:*** The Austroads suggested that during and after drug tapering and withdrawal, commercial drivers are no longer eligible for a conditional license unless an experienced specialist claims an acceptably low risk of seizure-induced crash. If reducing AED dose is only due to the occurrence of dose-related adverse events and is unlikely to cause a seizure, the patient can continue driving.^37^ The FMCSA guideline indicated that in the case of withdrawal from AEDs, at least an 8-year SFP from the time of drug cessation is recommended.^47^ The Canadian guideline accepted a 6-months SFP as an appropriate interval after AED withdrawal and if seizures recur, another 6-month SFP is enough if the patient has initiated a previously effective treatment regime and the treating physician indicates that further seizures are improbable.^58^

***Recommendation:*** Reassessment for a conditional license should be considered 10 years after AED withdrawal. If seizures recur, a 6-month SFP after resuming the previous therapeutic regime should be considered. Patients who experience the side effects of AEDs should refrain from driving during dose reduction of the responsible AED, until the side effects improve. When changing from one medication to another, a 6-month SFP after withdrawal from the first drug should be considered.

**After epilepsy surgery**

Regaining the permission to drive after epilepsy surgery can significantly improve the QOL of epileptic patients.^77^ Preoperative driving history, presurgical intracranial EEG monitoring and favorable surgical outcome (Engel class 1 and 2) are reported to be significant predictors of postoperative driving outcomes^78^ and are associated with regaining driving permission.^79^^-^^81^ However, some authors have shown that although better surgical outcomes are associated with regular active driving, no significant change has been observed after surgery in those patients who drive.^82^ It has been reported that individuals who have been seizure free for at least 8 years after surgery have an annual recurrence risk of ≤ 2% with a further risk reduction of ≤ 1% after 10 years.^47^

Moreover, tapering and discontinuation of AEDs following surgery accompany variable rates of seizure recurrence and occur in about one third of patients or more depending on the duration of the follow-up period.^79^^,^^83^^-^^85^ Therefore, if tapering is planned, the possibility of recurrence of seizures and the resulting risks should be considered^86^ and the suggested approach is the same as that of medication withdrawal in cases of controlled seizures following medical therapy.

***Private drivers:*** The recommended SFP before driving is determined as 6^47^^,^^58^ and 12 months^37^^,^^52^ following surgery. The important point is the necessity to reinvestigate patients for possible complications of surgery particularly visual field problems, which might affect the fitness to drive. Visual field deficit is a frequently observed adverse effect of medial temporal surgery and has been shown to cause prohibition of driving in 4%-50% of the patients.^87^ Since subjective symptoms and bedside visual field testing do not have the appropriate sensitivity to detect visual field deficits, considering standard perimetry before confirmation of the fitness to drive seems reasonable.^88^ Current guidelines have not suggested any approach for patients who only experience auras after surgery. In a study by Fairclough et al., it has been reported that although patients with aura after epilepsy surgery had a higher risk of seizure recurrence than those who are seizure-free, they may be allowed to drive since the risk is less than 20%.^89^

***Recommendation: ***Reassessment for issuing a conditional license should be considered in patients who have experienced a 6-month SFP with special attention to possible complications of surgery.

***Commercial drivers:*** The Austroads suggested the default standard of a 10-year SFP and related previously mentioned EEG criteria before license renewal considering that driving is no longer allowed in the case of any AED withdrawal.^37^ The Canadian guideline^58^ recommended a 5-year SFP and other guidelines did not address this issue.

***Recommendation:*** Reassessment for issuing a conditional license should be considered in patients who have experienced a 10-year SFP with special attention to possible complications of surgery.

**Seizure causing a crash**

Although most guidelines did not directly address seizures leading up to crashes, Austroads has cautiously recommended that patients who have had a crash or have lost control of the vehicle as a consequence of a seizure, should not be allowed to drive until after a 12-month SFP for private drivers and a 10-year SFP for commercial drivers. EEG criteria should also be met for commercial drivers.^37^

**Psychogenic non-epileptic seizure (PNES)**

It is estimated that about 20% of patients referred to epilepsy clinics for refractory seizures have PNES.^90^ However, this issue is not commonly addressed by current guidelines and little is known about the risk of driving in these patients due to lack of sufficient standard studies. Surveys have been conducted in 3 countries to assess expert opinion about the validity of approach to driving in patients with PNES. The results are however controversial. Three common answers have been addressed in studies assessing expert opinion in Germany and the United States. About half of the FMCSA experts suggested the same restrictions as those for epileptic patients followed by the suggestion of no restrictions in about one third of experts and individualized decision-making in others.^91^ Most of the German experts however preferred individualized decision-making while others suggested the same rules that were applied to epileptic patients.^92^

DVLA has suggested a license issue when medical reports confirm that behavioral disturbances have been satisfactorily controlled. Nevertheless, about 40% of English experts did not suggest driving restriction in patients with PNES and others personally recommended different time intervals for refraining from driving or accepted the DVLA guideline.^93^ Although current studies are not enough to draw a definite conclusion, it could be recommended that patients with pure PNES undergo a thorough evaluation regarding behavioral problems by an expert psychiatrist and renewing their license be considered after complete recovery. However, if any doubt exists about the possibility of co-existence of epilepsy and PNES, considering the previously mentioned rules for epileptic patients in addition to a complete psychiatric evaluation is recommended.^94^

**Practical recommendations for neurologists**

***Reassessment intervals*** *:* It is generally recommended that after the period of driving prohibition, the license be reviewed after 1 year and then within 5-year intervals. For seizures that do not influence driving ability or seizures that occur exclusively during sleep, the reassessment should be considered annually for the first 5 years. After this period, no specific assessment is necessary.^53^

***AED selection*** *: *AEDs might have undesirable side effects some of which such as drowsiness, dizziness, blurred vision, diplopia, and cognitive disturbances could result in serious deficits in driving abilities.^49^ Similar to other epileptic patients, the mainstay of treatment should be monotherapy with a selective AED with the least effective dose and minimum side effects. Consideration of probable drug interactions, measurement of serum drug level, and education of patients to report possible side effects could be helpful. When dose reduction or drug switching is planned, previously mentioned recommendations should be considered. 

**Necessary advice for patients**

***Reporting seizures*** *:* Fear of loss of driving license is among the reasons that epileptic patients might conceal their disease.^95^ In order to respect patients' rights, reporting the diagnosis of epilepsy or seizure recurrence by the clinicians is not mandatory in most parts of the world^96^^,^^97^ and it has been shown that compulsory reporting has no significant effect on the accident rate of epileptic patients.^98^^,^^99^ Nevertheless, ethically, the risk of driving for patient and public safety is also important. Deciding whether or not a compulsory report is required is based on governmental rules, but every clinician should present a complete explanation of possible risks of driving to the patients and encourage self-reports.^49^^,^^100^^-^^102^

***Avoiding trigger factors*** *: *Many epileptic patients report more frequent seizures during emotional stress, sleep loss, hyperventilation, consumption of alcohol or certain drugs, and menstrual cycle.^49^^,^^103^

In addition, many different and specific stimulating factors have been reported in reflex epilepsies.^103^ Educating patients to avoid driving in situations that make them more vulnerable to seizures and avoidance of specific trigger factors such as looking at light flashes, eating, somatosensory stimulation, and thinking during driving in patients with reflex epilepsy might reduce the risk of imminent seizures.

***Drug adherence*** *:* Poor adherence to AEDs has been reported in approximately one third of patients with epilepsy and is one of the main causes of seizure recurrence.^104^^-^^107^ It is also associated with a higher occurrence of car accidents.^108^^,^^109^ Patients should be educated on regular medication use and doctor consultation in the case of probable barriers to adherence.

## Conclusion

Permission to drive has a significant influence on the QOL of epileptic patients. However, decision-making in this regard is difficult due to the need to balance possible safety risks with patients’ rights. Different protocols have been postulated by jurisdictions all over the world, but some countries do not have a detailed guideline for these patients. Preparing comprehensive fitness to drive guidelines by all countries, updating them based on new evidences, and a general effort by experts to provide a common international and detailed guideline might prevent wrong or unnecessary individualized decision-makings and improve the QOL of epileptic patients. The recommendations of this manuscript are based on expert opinion and cannot be legally cited.

## Notes:

**How to cite this article:** Tabrizi N. Fitness to drive in seizure and epilepsy: A protocol for Iranian clinicians. Iran J Neurol 2019; 18(4): 159-71.
